# Heatwave-related variations in psychiatric consultations and admissions: a time-series analysis

**DOI:** 10.3389/fpsyt.2026.1803114

**Published:** 2026-05-18

**Authors:** Fabian M. Wedmann, Francesca Cella, Bart Toonen, Guenter K. Schiepek, Klaus Eisendle, Andreas Conca

**Affiliations:** 1 Psychiatrie und Psychotherapeutische Medizin, Bolzano Hospital: Ospedale di Bolzano, Bozen, Italy; 2 University Hospital of Psychiatry, Psychotherapy and Psychosomatics, Paracelsus Medical University, Salzburg, Austria; 3 Department of Psychiatry, Psychotherapy and Psychosomatics, Institute of Synergetics and Psychotherapy Research, Christian-Doppler University Hospital, Paracelsus Medical University, Salzburg, Austria; 4 Directorate, Claudiana—College of Health Professions, Bolzano, Italy

**Keywords:** climate change, heatwaves, psychiatric admissions, psychiatric consultations, time series analyses

## Abstract

**Background:**

Heatwaves are becoming increasingly frequent and intense across Europe, posing significant risks to physical and mental health. Emerging evidence suggests that prolonged exposure to high temperatures may exacerbate psychiatric symptoms and increase the demand for acute mental health services.

**Objectives:**

This study examined the relationship between extreme heat events and psychiatric service utilization in Bolzano, Italy, by analyzing emergency psychiatric consultations and acute psychiatric admissions across three non-consecutive years.

**Methods:**

A retrospective observational analysis was conducted using daily psychiatric consultations in the Emergency Department (ED) and daily admissions to acute psychiatric wards from 2013, 2018, and 2023. Meteorological data were obtained from the provincial environmental agency. Time-series analyses employed ARIMA models, incorporating daily minimum and maximum temperatures, tropical nights, and a cumulative heatwave index (n_hot_htwv). Model selection was based on BIC, and the effect of exogenous temperature variables was evaluated through changes in AIC. Residual diagnostics guided the inclusion of weekly seasonal dummy variables.

**Results:**

Non-seasonal ARIMA models with day-of-week dummies provided the best fit for both consultations and admissions. Adding the cumulative heatwave variable (n_hot_htwv) consistently improved model fit across all years, whereas minimum and maximum temperatures alone did not. Heatwave duration emerged as a more sensitive predictor of psychiatric service utilization than isolated temperature peaks. No evidence of yearly seasonality was found, and residual diagnostics supported the robustness of models including weekly dummy variables.

**Conclusion:**

Heatwaves are associated with increased psychiatric consultations and hospital admissions in Bolzano, with cumulative heat exposure representing a critical determinant. These effects cannot be explained solely by seasonal patterns, suggesting an independent climatic influence. Given the projected rise in heatwave intensity and duration, mental health services should incorporate climate-responsive planning and early-warning strategies.

## Introduction

Climate change is among the most pressing global challenges of the 21st century, with heatwaves emerging as increasingly frequent, intense, and prolonged events (1, 2). These phenomena pose serious risks to human health, particularly for vulnerable populations such as the elderly, children, and individuals with comorbidities (3, 4).

Among these groups, children represent a particularly sensitive population. Recent evidence shows that extreme heat is associated with increased sleep disturbances, irritability, and emergency visits during heatwave periods, reflecting reduced thermoregulatory capacity and limited behavioral adaptability (5). Similarly, individuals affected by severe mental illness appear to be disproportionately vulnerable to heat-related stress. During extreme events such as the 2021 Vancouver heat dome, analyses of community deaths revealed markedly increased mortality among patients with schizophrenia, illustrating how extreme heat can exacerbate existing vulnerabilities in certain psychiatric populations (6).

Beyond these specific high-risk groups, increased vulnerability to heat exposure is also reflected at the level of mental health service utilization. Over the past two decades, epidemiological studies using time-series and case-crossover designs have consistently reported short-term increases in psychiatric emergency visits and hospital admissions during periods of elevated ambient temperature (7–9). These temperature-related increases have been observed across a broad range of diagnostic groups, including mood disorders, substance use disorders, psychotic conditions, and dementia-related disorders, suggesting a system-wide impact of heat exposure on psychiatric care demand (10–12).

Heatwaves are commonly defined as periods of several consecutive days in which temperatures exceed locally specific climatic thresholds, typically based on high percentile cutoffs of historical temperature distributions This framing is consistent with international recommendations, which describe heatwaves as persistent periods of unusually hot weather lasting at least two to three days relative to local climatological norms, often determined using thresholds in the 90th or 95th percentile range. Such definitions capture both the absolute and relative extremity of heat in a given region, providing a robust basis for identifying periods of substantial thermal stress (13).

Importantly, recent research emphasizes that the cumulative duration of heat exposure plays a more critical role than isolated single-day temperature spikes, particularly for mental health outcomes. Sustained heat over multiple days leads to progressive physiological strain, sleep disruption, autonomic dysregulation, dehydration, and impaired thermoregulation—mechanisms that are more likely to precipitate psychiatric destabilization than brief, transient peaks in temperature (14).

The impact of heatwaves is further amplified when high daytime temperatures coincide with limited nocturnal cooling, such as during “tropical nights,” when nighttime temperatures remain above 20 °C. Under these conditions, the body is deprived of essential overnight recovery, magnifying the adverse effects of prolonged heat exposure on both physiological and psychological well-being (6, 15, 16). Sustained thermal stress not only exacerbates pre-existing medical conditions but also increases the incidence of heat-related illnesses and fatalities, placing substantial pressure on healthcare and emergency services.

Recent research has highlighted the neurobiological mechanisms through which heat stress impacts mental health (17, 18). Elevated ambient temperatures can disrupt thermoregulation, increase cortisol levels, and suppress parasympathetic activity, contributing to anxiety, mood instability, and sleep disturbances (19). Moreover, extreme heat may induce oxidative stress and neuroinflammation, impairing hippocampal neurogenesis and cognitive function (19–21). These biological pathways help explain the observed rise in psychiatric emergencies during periods of extreme heat and underscore the need for targeted public health interventions.

Given the regional variability of heatwaves, localized assessments are essential. Bolzano, capital of South Tyrol in northern Italy, presents a unique microclimate shaped by its Alpine basin geography. With over 300 sunny days annually and frequent summer temperatures exceeding 30 °C, the city experiences numerous tropical nights. Since the 1980s, average annual temperatures have risen by 2 °C, with summer temperatures increasing by up to 3 °C (22).

The region has experienced a significant increase in temperatures over recent decades: average annual temperatures in South Tyrol have risen by approximately +2.2 °C since 1980, reflecting a statistically significant warming trend of +0.5 °C per decade, with 2022–2024 being the warmest years on record.

At the urban scale, Bolzano shows a long-term warming trend of +0.43 °C per decade (1970–2024), further illustrating the intensification of heat-related climatic conditions in the area (22, 23).

To assess the local impact of heat on mental health, we conducted a retrospective analysis correlating psychiatric emergencies and acute admissions at Bolzano’s central hospital with regional climate data.

## Methods

### Study design and objectives

This retrospective observational study aimed to investigate the relationship between extreme heat events and psychiatric service utilization in Bolzano, Italy. Specifically, we assessed whether the number of psychiatric consultations in the Emergency Department (ED) and hospital admissions to acute psychiatric wards increased during heatwave periods or in the days immediately following them, compared to baseline trends.

### Data sources and inclusion criteria

Clinical data were extracted from the hospital records of San Maurizio Hospital in Bolzano with a catchment area of 242.000 inhabitants for the years 2013, 2018, and 2023. The years 2013, 2018, and 2023 were selected to represent a 10-year interval with 5-year spacing. The year 2013 was specifically included because it marked a structural reorganization in the collaboration between the psychiatric service and the Emergency Department, as well as changes in outpatient treatment pathways. These organizational shifts resulted in a more standardized and consistent referral and consultation process, making 2013 the earliest year for which ED psychiatric consultation data are fully comparable to subsequent years. The inclusion of 2018 and 2023 allowed us to examine trends across a decade while capturing the progressive increase in extreme-heat events in the region.

Two outcome variables were considered:

- Daily number of psychiatric consultations performed in the ED
- Daily number of psychiatric admissions to acute psychiatric wards (SPDC)

Although psychiatric consultations are performed across all hospital departments, only those conducted in the ED were included in the analysis. This choice was based on the fact that ED consultations consistently represent acute psychiatric presentations with clear temporal proximity to environmental stressors. In contrast, consultations in other departments may be delayed, non-urgent, or related to pharmacological monitoring, making them less suitable for time-sensitive analysis.

Admissions were included regardless of time of day or mode of referral. Based on the Italian community model of Psychiatry, in the South Tyrol region, psychiatric admissions are not constrained by bed availability (n=26), ensuring that all (per)acute patients requiring hospitalization are admitted without deferral or transfer to other facilities.

### Climate data and heatwave definition

Meteorological data were provided by the Provincial Agency for Environment and Climate Protection (Landesagentur für Umwelt und Klimaschutz). Variables included:

- Daily mean, maximum (temp.max), and minimum (temp.min) temperatures
- Number of days with maximum temperature >35 °C
- Number of tropical nights (minimum temperature >20 °C)

The threshold of 35 °C used in this study does not derive from health indicators but reflects the official climatological definition of heatwaves provided by the regional meteorological authority (Amt für Meteorologie und Lawinenwarnung). This definition is based on long-term regional climatological analyses and is used operationally for weather warnings in Alpine areas. According to this authority, a heatwave is defined as a period of at least three consecutive days with maximum temperatures exceeding 35 °C.

Because heatwave definitions vary substantially across Europe depending on regional microclimate and local thermal norms, using the locally validated meteorological threshold avoids applying criteria developed for different climatic contexts (e.g., Mediterranean or continental regions). In our Alpine setting, temperatures above 35 °C are rare and represent an extreme and atypical deviation from usual summer conditions, which justifies their use as an indicator of extraordinary heat stress independent of health outcomes. Although such events remain infrequent, observational data show a gradual upward trend in their occurrence, underscoring their growing relevance as a marker of climatic extremes. Based on this operational definition, we constructed the variable n_hot_htwv, which captures the number of consecutive days above the 35 °C threshold; values ≥3 indicate occurrence of a heatwave event.

### Preliminary analysis

A preliminary inspection of the autocorrelation function (ACF) and the partial autocorrelation function (PACF) for cons and ric showed the possible presence of seasonality, with a period of 7 days. This seasonality was taken into account in the subsequent Autoregressive Integrated Moving Average (ARIMA) analyses. Due to the nonconsecutive years in the data, yearly seasonality was not analyzed. All subsequent analyses were applied to each year separately – not to the full dataset.

### ARIMA analysis

The ARIMA model for time-series analysis combines autoregressive (AR), integrated (I) and moving average (MA) models, where the integrated part allows for the analysis of nonstationary time-series by differencing the data (24). The ARMA part is based on the underlying assumption that in a linear time-series there are two separate contributions to a variable’s value. The AR part consists of a weighted regression on the variable’s past values, and can be used to analyze the occurrence of persistent trends within the data. The MA part consists of a weighted regression on the past forecast errors, and thereby describes the response of the system to sudden shocks. Besides a regression on its own values, some ARIMA models also allow for the inclusion of exogenous variables to predict the value of a time-series’ value (25).

The ARIMA analyses consisted of two steps. The first step used the time-series data for cons and ric, without any temperature data. The aim of this step was to find the ARIMA model that gave the best model fit, as measured by the Bayesian Information Criterion (BIC), using the auto.arima function from R’s forecast package (26). The following models were used: (A) models without any seasonal dummies or seasonal ARIMA components; (B) models without any seasonal dummies, but with seasonal ARIMA components and (C) models with seasonal dummies and seasonal ARIMA components. A comparison of the BICs resulted in a best-fitting model for each year separately.

The aim of the second step was to analyze the change in the model fit, as measured by the Akaike Information Criterion (AIC), after including an exogenous temperature variable (27). The AIC puts a lower penalty on the inclusion of additional variables than the BIC. Since the aim of this step was to analyze the influence of adding the information of the exogenous variable – not to find the model with the lowest number of parameters – the BIC was not used here. The following exogenous temperature-variables were used: temp.min, temp.max and n_hot_htwv. A decrease in the AIC indicated a better model fit.

## Results

### ARIMA analysis, first step

**Table 1** shows the BIC results for cons, using auto.arima. Model A contains no seasonal dummies and no seasonal ARIMA components. Model B contains no seasonal dummies but is allowed to contain seasonal ARIMA components. If its BIC values are the same as for Model A, this indicates that auto.arima did not use the seasonal part (i.e. the optimal model is the same as Model A). Model C contains seasonal dummies and is allowed to contain seasonal ARIMA components. These results show that, for 2013 and 2018, the models without any seasonal dummies or seasonal ARIMA components give the best model fits. This implies that in these two years, the consultations data does not show a large amount of seasonality. In 2023, however, a model with seasonal dummies and/or seasonal ARIMA components gives the best fit. Further inspection of that model shows that all ARIMA parameters (p, d, q, P, D, Q) have a value of zero, meaning no autoregressive, integrative or moving average components, seasonal nor nonseasonal. This implies that the seasonal dummy variables contain all the necessary information.

**Table 1:** BIC values of several ARIMA models for cons data.

Year	Model A	Model B	Model C
2013	1296.7	1296.7	1319.2
2018	1343.4	1343.4	1369.9
2023	1487.1	1477.6	1439.1

**Table 2** shows the results for ric, using the same types of model. These results show that the number of admissions is best modelled with Model C – the models that contain seasonal dummies and are allowed to contain seasonal ARIMA components. Further inspection of those models show ARIMA components (p, d, q, P, D, Q) of (0, 0, 0, 0, 0, 0), (0, 0, 0, 0, 0, 0) and (0, 1, 1, 0, 0, 0) for 2013, 2018 and 2003 respectively. This implies that for 2013 and 2018, the inclusion of dummy variables alone is sufficient to model the seasonal component. For 2023, there still remains a moving average (MA) component and the data is best modelled after differencing the data once. This suggest some nonstationarity as well as some amount of short lasting change after a shock.

**Table 2:** BIC values of several ARIMA models for ric data.

Year	Model A	Model B	Model C
2013	1342.6	1342.6	1335.9
2018	1347.0	1343.0	1338.8
2023	1401.7	1399.2	1396.4

### ARIMA analysis, second step

The second step uses the models with the best fit from step one. For cons, these are Model A, Model B and Model C for 2013, 2018 and 2023 respectively. For ric, these are Model C, for each year. The corresponding AIC values are, for cons: 1288.9, 1335.6 and 1407.9, and for ric: 1304.7, 1307.6 and 1365.2. **Table 3** shows the AIC values for cons, after addition of an exogenous temperature variable. For 2013 this shows a worse model fit after addition of temp.min or temp.max, but a better model fit after addition of n_hot_htwv. For 2018, an improvement of the model fit is observed for all three variables, with the largest decrease in AIC for n_hot_htwv. For 2018 temp.min and temp.max result in a worse model fit and n_hot_htwv to a better one. In short, the addition of n_hot_htwv leads to the best model fit for cons in all three years.

**Table 3:** AIC values of the best performing ARIMA model for cons after addition of temperature.

Year	temp.min	temp.max	n_hot_htwv
2013	1290.6	1289.8	1285.5
2018	1332.5	1333.9	1330.5
2023	1409.9	1409.9	1401.9

**Table 4** shows the AIC values for ric, after addition of an exogenous temperature variable. For 2013 this shows a slightly better model fit after addition of temp.min, a slightly worse model fit after addition of temp.max and a better model fit after addition of n_hot_htwv. For 2018 and 2023, only the addition of n_hot_htwv leads to a better model fit.

**Table 4:** AIC values of the best performing ARIMA model for ric after addition of temperature.

Year	temp.min	temp.max	n_hot_htwv
2013	1303.1	1305.1	1298.4
2018	1307.6	1308.4	1303.1
2023	1365.4	1365.8	1361.0

### Residual analysis

Residual analyses for each model showed that for most models the residuals were not normally distributed. See Appendix 1. Only models that included dummy variables for day of the week showed normally distributed residuals, although not for all days. A logarithmic data-transform did not improve the results. This nonnormality could possibly compromise the values for AIC and BIC, and the validity of confidence intervals for the model parameters. Therefore, it would be preferred to base results only on analyses with dummy variables. This is already the case for the models for ric, because for this variable the best performing models were the models with dummies. However, for cons this is not the case. It was therefore decided to do an additional analysis for cons that only used models with dummy parameters for day of the week. These showed AIC values of 1284, 1333 and 1402 for 2013, 2018 and 2023 respectively. Compared with the results without temperature data, showing AIC values of 1288, 1339 and 1408, this is an improvement.

### Final remarks

Based on BIC values and normality of the residuals, both cons and ric, without temperature are best modelled by nonseasonal ARIMA-models with dummy variables for the day of the week. Adding the exogenous variable n_hot_htwv resulted in a better model fit for cons and ric.

## Discussion

Our study demonstrates a clear trend between heatwaves and psychiatric consultations as well as emergency admissions through the central emergency department of a large regional hospital across three distinct, non-consecutive years. These findings are consistent with previous epidemiological investigations reporting increased psychiatric service utilization during periods of elevated ambient temperatures (28, 29). In line with the observational nature of the data, this trend should be interpreted as an association rather than evidence of a causal relationship.

The observed association aligns with neurobiological evidence suggesting heightened vulnerability among psychiatric patients when exposed to high environmental temperatures.

Heat stress can trigger a pronounced neuroinflammatory response in the CNS via microglial activation and release of proinflammatory cytokines (IL 1β, IL 6, TNF α, IL 18) (30). These mediators inhibit neurotrophic signaling, particularly BDNF-dependent pathways, and reduce markers of hippocampal neurogenesis such as DCX. Thermosensitive receptors (e.g., TRPV4), oxidative stress, and mitochondrial dysfunction further compromise neuronal precursor survival (31). Given the hippocampus’ role in stress regulation and emotional processing, impaired neurogenesis may increase vulnerability to depressive and anxiety-related symptoms (19, 21, 32, 33).

Vulnerable clinical groups, including individuals with schizophrenia, exhibit disproportionately elevated morbidity and mortality during extreme heat events; during the 2021 Canadian heat dome, people with schizophrenia accounted for a markedly increased share of heat-related deaths despite their low population prevalence. Moreover, psychotropic medications—such as antipsychotics, antidepressants, benzodiazepines, and lithium—can exacerbate heat vulnerability by impairing thermoregulation, reducing thirst perception, altering sweating, and increasing the risk of dehydration-related toxicity (17, 34, 35). Heat exposure also disrupts sleep and circadian rhythms, leading to heightened HPA axis activation, emotional dysregulation, and worsening of mood, anxiety, and psychotic symptoms—an effect well documented in recent mechanistic investigations of extreme heat and mental health (17, 33, 36).

Heatwaves occur relatively infrequently throughout the year, limiting the number of observations and complicating statistical analysis. Unlike other studies, we deliberately refrained from applying a linear model to avoid oversimplification and to account for natural fluctuations in consultation and admission rates (e.g., public holidays, typical “admission days” such as Mondays and Fridays). This approach allowed us to capture subtle variations hypothesized to occur during heatwaves.

Seasonal variations in psychiatric conditions must also be considered. For instance, manic episodes in bipolar disorder tend to rise during spring and summer, likely due to increased daylight exposure (37, 38). However, the absence of a significant difference in overall monthly admission rates suggests that seasonal patterns alone cannot explain the observed correlations.

A particularly noteworthy finding concerns the role of heatwaves compared to isolated temperature measurements. The indicator used in this study, n_hot_htwv—capturing consecutive days above a defined threshold—proved more sensitive than isolated daily minimum or maximum temperatures. This supports evidence that prolonged heat exposure and lack of nocturnal cooling exert stronger effects on mental well-being than single temperature peaks.

Geographical characteristics must also be considered. Nearly one fourth of our hospital’s catchment area lies at higher altitudes (500–1500 m), where lower temperatures and nocturnal cooling act as a natural protective factor, likely attenuating heat-related stress (**Supplementary Table 1**) (39, 40).

### Strengths

Key strengths include a sophisticated statistical approach mitigating bias inherent in retrospective designs, selection of non-consecutive years to exclude systemic outliers, and consideration of heatwave duration rather than isolated temperature peaks. While we hypothesized a lagged increase in admissions following heatwave onset, this could not be demonstrated with our methodology. Future research should employ impulse-response analysis to explore delayed effects.

### Limitations

Major limitations include the absence of individual-level diagnostic and demographic data, which precludes conclusions about differential vulnerability across specific psychiatric disorders or population subgroups (e.g., age or gender). As a result, stratified analyses could not be conducted to determine whether heat-related increases in psychiatric emergency utilization are evenly distributed across patient groups or more pronounced in specific subpopulations. This limitation reflects the study’s deliberate reliance on anonymized aggregate hospital data and the resulting lack of access to individual-level information.

Heatwaves are also correlated with other meteorological phenomena—such as ozone, nitrogen oxides, and drought periods—that may themselves act as independent risk factors for psychiatric destabilization. Although daily maximum and minimum temperatures were available, the present analyses did not specifically examine intra-day temperature variability, which may represent an additional dimension of heat exposure. Furthermore, important confounders such as air pollution, holiday-related fluctuations in population density, and changes in service organization were not measured and may partially contribute to the observed patterns.

A major methodological limitation is the inclusion of non-consecutive years, which may distort underlying temporal trends and reduce comparability across observation periods. In addition, the selection of these years was constrained by the availability of complete, manually retrievable datasets, and preliminary statistical assessments showed substantial differences in ARIMA parameters across years, suggesting potential non-stationarity that would have complicated the construction of a unified multi-year time series. Despite these constraints, the primary public health implication remains: anticipating increased psychiatric service demand during heatwaves.

Finally, the heatwave threshold of 35 °C used in this study reflects meteorological criteria established in collaboration with the regional warning center rather than being derived from health-outcome-based metrics. As temperatures in Bozen/Bolzano increasingly reach or exceed this level, this threshold may require re-evaluation in future analyses, representing an additional limitation of the present study.

## Conclusion

Our analysis demonstrates a consistent association between heatwaves and increased psychiatric consultations and emergency admissions. Heatwave duration appeared more informative than isolated temperature peaks, and these patterns were not attributable to seasonal variation alone.

Although the study design and unmeasured confounding factors limit causal interpretation, the findings indicate that prolonged heat coincides with higher psychiatric service demand. From a policy perspective, these results support enhancing heatwave preparedness within mental-health and emergency services, including early-warning coordination and appropriate resource allocation during periods of extreme heat.

## Data Availability

The raw data supporting the conclusions of this article will be made available by the authors, without undue reservation.
