# Supplementary figures and images for: Heatwave-related variations in psychiatric consultations and admissions: a time-series analysis
Source: Front Psychiatry. 2026 May 18;17:1803114. doi: 10.3389/fpsyt.2026.1803114 (PMC13222980; doi:10.3389/fpsyt.2026.1803114)

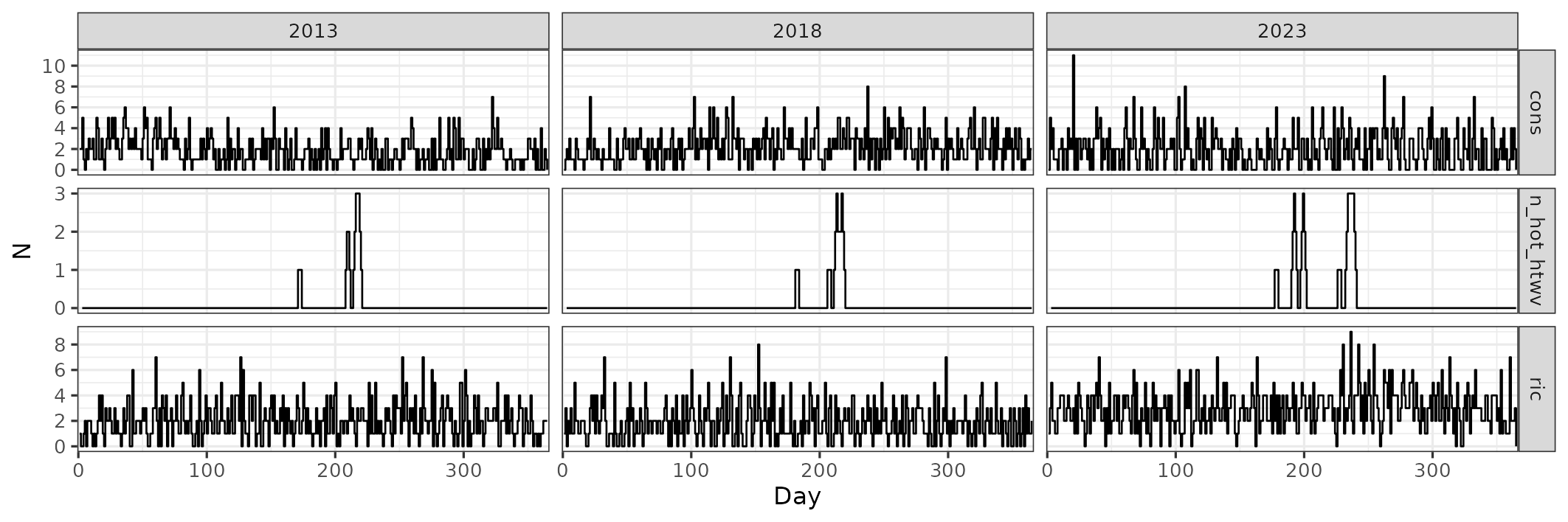

Supplement: Supplementary file 1 [file Image1.tiff]

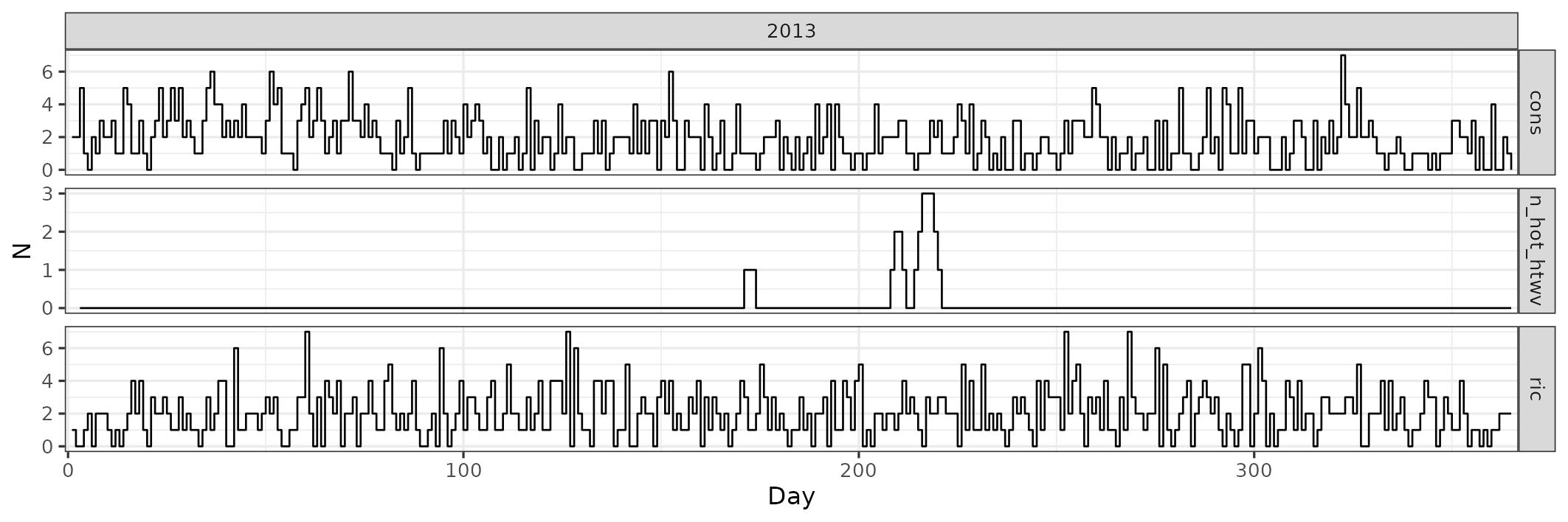

Supplement: Supplementary file 2 [file Image2.tiff]

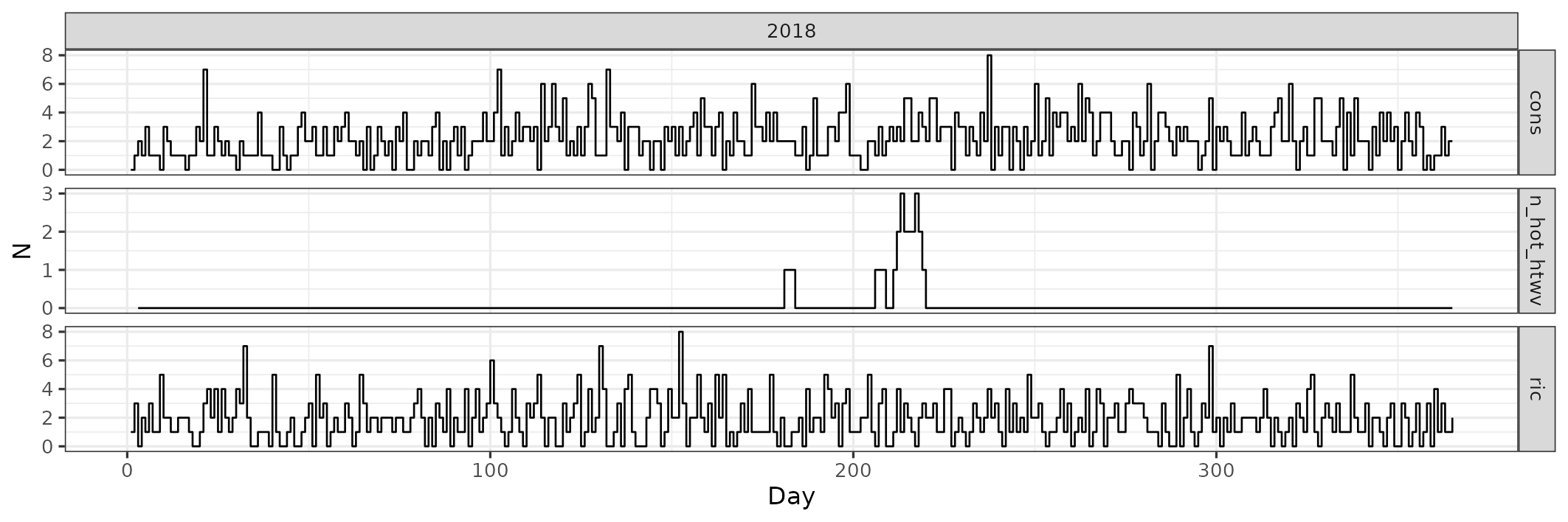

Supplement: Supplementary file 3 [file Image3.tiff]

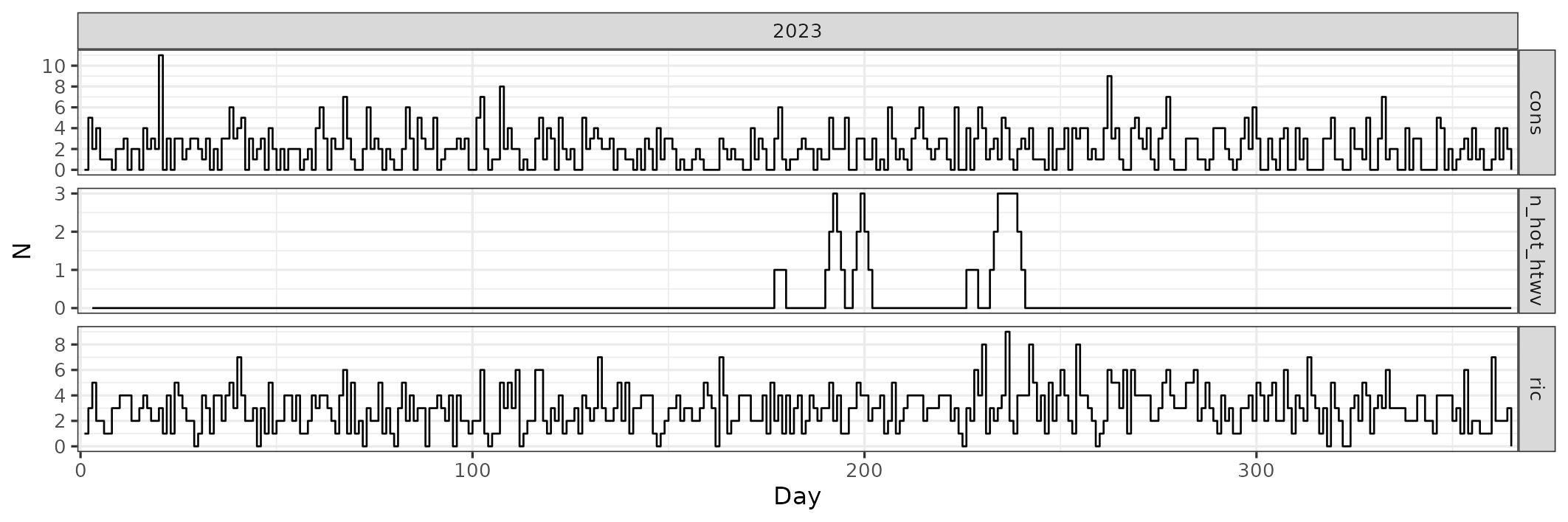

Supplement: Supplementary file 4 [file Image4.tiff]
