# Supplementary material for: Heatwave-related variations in psychiatric consultations and admissions: a time-series analysis
Source: Front Psychiatry. 2026 May 18;17:1803114. doi: 10.3389/fpsyt.2026.1803114 (PMC13222980; doi:10.3389/fpsyt.2026.1803114)
Supplement: Supplementary file 5 [file Table1.docx]

# Catchment Area – Municipalities, Population, Elevation (sorted by altitude class)

| Municipality | Population | Elevation (m) | Altitude class |
| --- | --- | --- | --- |
| Bolzano | 106 463 | 262 | Valley floor |
| Laives | 18 659 | 255 | Valley floor |
| Appiano | 14 963 | 415 | Valley floor |
| Caldaro | 8 267 | 425 | Valley floor |
| Termeno | 3 399 | 276 | Valley floor |
| Salorno | 3 827 | 224 | Valley floor |
| Egna | 5 535 | 214 | Valley floor |
| Ora | 3 883 | 242 | Valley floor |
| Terlano | 4 945 | 248 | Valley floor |
| Andriano | 1 046 | 274 | Valley floor |
| Montagna | 1 738 | 497 | Valley floor |
| Bronzolo | 2 793 | 238 | Valley floor |
| Vadena | 1 104 | 243 | Valley floor |
| Cornedo all’Isarco | 3 425 | 290 | Valley floor |
| Magrè sulla Strada del Vino | 1 320 | 241 | Valley floor |
| Cortina sulla Strada del Vino | 662 | 212 | Valley floor |
| Cortaccia sulla Strada del Vino | 2 232 | 333 | Valley floor |
| Fiè allo Sciliar | 3 710 | 880 | Mid-mountain |
| Sarentino | 7 266 | 970 | Mid-mountain |
| Tires | 1 037 | 1028 | Mid-mountain |
| Castelrotto | 7 079 | 1060 | Mid-mountain |
| San Genesio | 3 018 | 1100 | Mid-mountain |
| Meltina | 1 731 | 1140 | Mid-mountain |
| Renon | 8 204 | 1154 | Mid-mountain |
| Nova Levante | 1 945 | 1182 | Mid-mountain |
| Aldino | 1 604 | 1225 | Mid-mountain |
| Ortisei | 4 741 | 1233 | Mid-mountain |
| Nova Ponente | 4 011 | 1357 | Mid-mountain |
| Santa Cristina | 2 035 | 1428 | Mid-mountain |
| Trodena nel Parco Naturale | 1 053 | 1127 | Mid-mountain |
| Anterivo | 397 | 1209 | Mid-mountain |
| Selva | 2 618 | 1563 | High-mountain |

**Totals and percentages by altitude class**

Total population (all municipalities): 234 710

Valley floor: 184 261 (78.5%)

Mid-mountain: 47 831 (20.4%)

High-mountain: 2 618 (1.1%)

Note: Population figures use the latest values compiled in our working table (ISTAT-derived for added municipalities); elevations refer to municipal centre heights.
