# Supplementary material for: Heatwave-related variations in psychiatric consultations and admissions: a time-series analysis
Source: Front Psychiatry. 2026 May 18;17:1803114. doi: 10.3389/fpsyt.2026.1803114 (PMC13222980; doi:10.3389/fpsyt.2026.1803114)
Supplement: Supplementary file 6 [file Table2.docx]

| year | temp_max (^°^C) | temp_min (^°^C) | n_hot_htwv | n_htwv |
| --- | --- | --- | --- | --- |
| 2013 | 18.4 (9.4*) | 8.3 (7.4) | 8 | 3 |
| 2018 | 19.4 (9.7) | 8.8 (7.7) | 8 | 2 |
| 2023 | 19.8 (8.9) | 8.9 (7.6) | 15 | 7 |

* Standard deviation

Table XX. Descriptive statistics of temperature.

temp_max – mean and standard deviation (between brackets) of daily maximum temperature; tem_min – mean and SD of daily minimum temperature, n_hot_htwv – number of days when temp_max exceeded 35 ^°^C; n_htwv – number of periods where three consecutive days had temp_max exceeding 35 ^°^C.

|  | Sunday | | Monday | | Tuesday | | | Wednesday | | Thursday | | Friday | | | Saturday | |
| --- | --- | --- | --- | --- | --- | --- | --- | --- | --- | --- | --- | --- | --- | --- | --- | --- |
| Year | cons | ric | cons | ric | | cons | ric | cons | ric | cons | ric | | cons | ric | cons | ric |
| 2013 | 1.5 (1.1*) | 1.1 (1.1) | 2.1 (1.6) | 2.5 (1.7) | | 2.2 (1.5) | 1.9 (1.2) | 2.0 (1.5) | 2.3 (1.6) | 1.7 (1.3) | 2.5 (1.5) | | 1.8 (1.4) | 2.2 (1.6) | 1.6 (1.5) | 1.5 (1.2) |
| 2018 | 1.9 (1.4) | 1.0 (0.9) | 2.3 (1.5) | 2.2 (1.4) | | 2.2 (1.6) | 2.2 (1.4) | 1.9 (1.2) | 1.8 (1.6) | 2.4 (1.6) | 2.1 (1.7) | | 2.5 (1.6) | 2.5 (1.6) | 2.3 (1.6) | 1.2 (1.1) |
| 2023 | 0.8 (1.1) | 2.1 (1.3) | 2.7 (1.6) | 2.9 (1.6) | | 2.7 (2.1) | 3.4 (1.5) | 2.9 (1.6) | 3.5 (1.7) | 2.0 (1.6) | 3.2 (1.7) | | 2.4 (2.0) | 3.2 (1.6) | 0.8 (1.1) | 2.2 (1.4) |

^* Standard deviation^

Table XX. Daily descriptive statistics of consultations and admissions.

Daily averages of the number of consultations (cons) and admissions (ric).
